# A Systematic Approach to Evaluate Patients Presenting With ST-Segment Elevation in Lead aVR: A Case Series

**DOI:** 10.7759/cureus.11800

**Published:** 2020-11-30

**Authors:** Willis Ko, Gina Hurng, Ruihai Zhou, Xuming Dai

**Affiliations:** 1 Cardiology, New York Presbyterian Queens, New York, USA; 2 Cardiology, Coney Island Hospital, Brooklyn, USA; 3 Cardiology, University of North Carolina at Chapel Hill School of Medicine, Chapel Hill, USA

**Keywords:** st segment elevation, st elevation myocardial infarction, global ischemia, coronary artery disease

## Abstract

ST-segment elevation (STE) in the lead aVR indicates global ischemia of the myocardium and is often associated with obstructive coronary artery disease (CAD). We report a serial of cases presenting with STE in aVR and diffuse ST depressions in more than six other leads as a common feature, but of different etiologies, i.e., severe anemia due to gastrointestinal bleeding; drug over-dose-induced vasospasm and tachycardia, and severe CAD involving distal left main and ostial right coronary arteries, which required specific management approaches. We categorize the possible causes of STE in aVR with or without diffuse ST depression ECG according to whether anticoagulation/antithrombotic agents are indicated, contra-indicated, and propose a systematic approach in evaluating and managing these patients.

## Introduction

In standard 12-lead electrocardiography (ECG), the augmented unipolar limb lead of the right arm (aVR) reflects the right upper portion of the heart, including the outflow tract of the right ventricle, basal portion of the septum, and the reciprocal aspect of the left lateral side of the ventricle covered by leads aVL, II, V5, and V6. Many clinical observations have shown that ST-segment elevation (STE; >0.5 mm) in lead aVR could predict the presence of significant left main coronary artery (LMCA) disease or triple vessel disease with high sensitivity and specificity [1]. The fourth Universal Definition of Myocardial Infarction appropriated the use of “ST-segment elevation in lead aVR with specific repolarization patterns, as a STEMI equivalent” [2]. The recently updated European Society of Cardiology guidelines for the management of non-STE acute coronary syndrome (NSTE-ACS) recommend immediate (<2 hours) invasive strategy for NSTE-ACS patients with “the presence of ST-segment depression >1 mm in six leads additional to ST-segment elevation in aVR and/or V1” [3]. In the era of using primary percutaneous coronary intervention (PCI) to treat STE myocardial infarction (STEMI) with an emphasis on timely reperfusion as reflected by first-device activation time, whether patients with STE in aVR should be considered as a STEMI equivalence with subsequent immediate activation of the cardiac catheterization laboratory (CCL) is a question of active discussion among emergency medicine and cardiology providers. Global ischemia of the myocardium, which is often reflected as STE in lead aVR and diffuse ST-segment depressions in other leads, could be a result of other etiologies, in particular, conditions in which it would be contraindicated to perform invasive CCL procedures. We present a series of cases, which in addition to highlighting the importance of acquiring enough clinical information in patients with STE in lead aVR to rule out potential conditions in which CCL procedures would be detrimental, also reinforces the importance of pursuing an invasive approach in a safe and timely manner for the right patients. We also propose a systematic strategy in evaluating and managing acutely ill patients with STE in aVR.

## Case presentation

Patient 1

A 66-year-old man with a history of morbid obesity, pre-diabetes mellitus, hypertension, and hyperlipidemia presented to the emergency room complaining of fatigue for approximately 24 hours. He had associated symptoms of orthopnea, decreased exercise tolerance, lightheadedness, and dizziness. He denied chest pain, palpitations, syncope and had no history of tobacco use or family history of cardiac conditions. Vitals showed tachycardia at 116 beats per minute and regular rhythm. The rest of his vitals were within normal limits. Physical examination revealed III/VI systolic murmur in the sternal border with radiation to the axilla.

**Figure 1:**
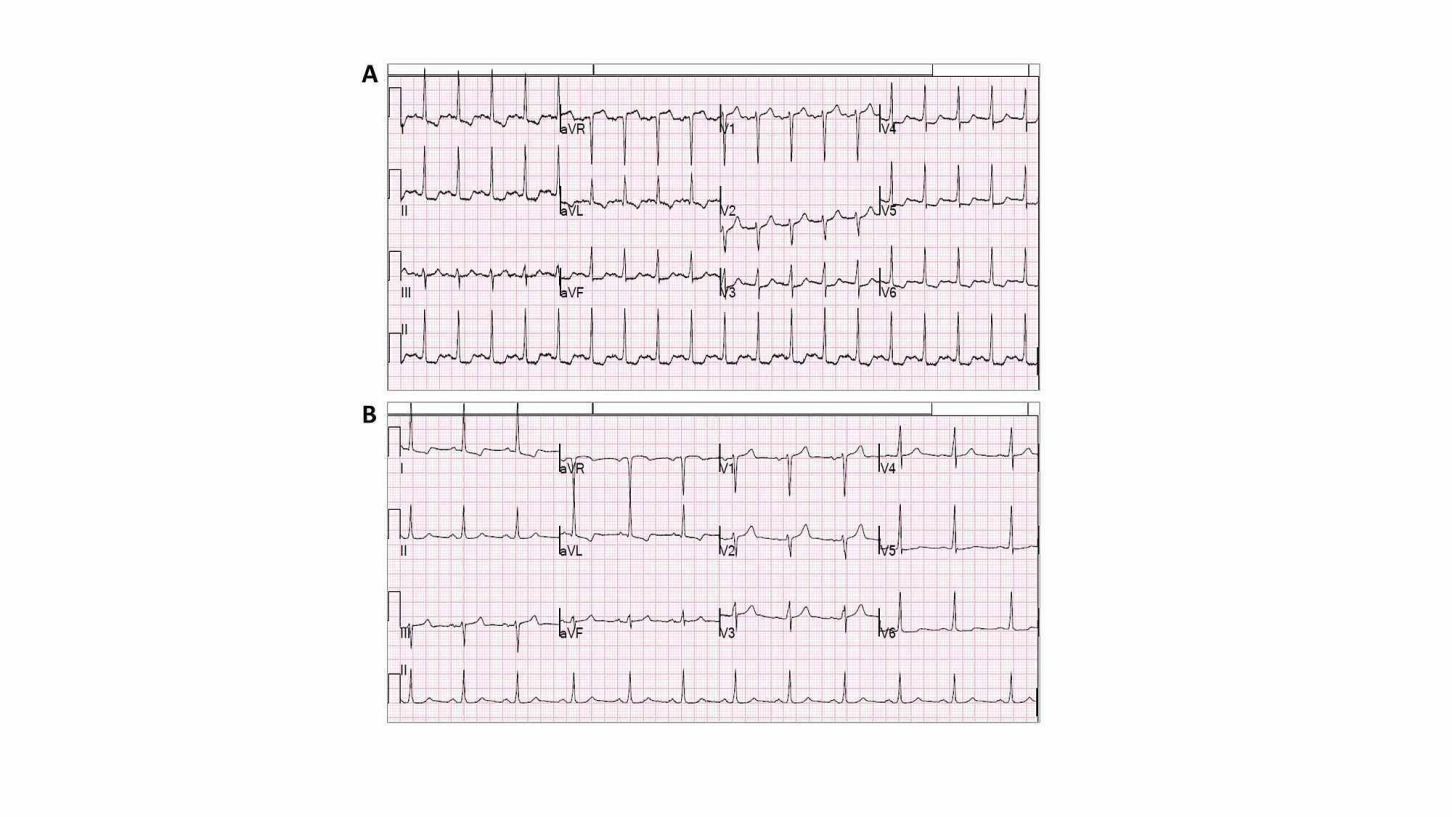
Twelve-lead ECGs of patient 1 (A) Presenting ECG in the ED revealed STE in aVR and V1, and horizontal ST depressions in leads I, II, aVF, V3-V6 associated with variable degrees of T-wave inversions in I and aVL. (B) Subsequent ECG after PRBC transfusion showed resolved STE in aVR and ST depressions in most of the leads. ECG: electrocardiogram, ED: emergency department, STE: ST-segment elevation, PRBC: packed red blood cells.

After obtaining an ECG from the emergency department (ED) team, the STEMI alert was activated, which automatically triggered CCL team activation. The cardiology team responded to the STEMI alert and evaluated the patient in the ED. The 12-lead ECG obtained at presentation revealed sinus tachycardia, 1-1.5 mm STE in aVR, and horizontal ST-segment depressions in I, II, aVF, V3-V6 associated with various degrees of T-wave inversions in these leads (Figure 1A). Despite the presence of concerning ECG abnormalities, the patient’s clinical presentation was atypical for an acute myocardial infarction with an overall relatively stable clinical status. Therefore, the CCL activation was cancelled with recommendations of obtaining additional information including a panel of laboratory tests, chest radiography, and observation in a telemetry unit with serial ECGs if his condition changes. His bloodwork revealed an initial troponin-T of 0.112 ng/ml (normal <0.03 ng/ml), hemoglobin of 4.9 g/dL, and a positive stool occult blood test. Severe anemia due to GI bleeding was considered to be the primary etiology for the patient’s presentation and abnormal ECG. After transfusion of 2 units of packed red blood cells with the subsequent response of hemoglobin to 6.5 g/dL, his troponin-T trended down to 0.097 ng/ml. He was subsequently found to have two arteriovenous malformations (AVM) with endoscopy, which were treated with electrocautery. His symptoms and ECG findings significantly improved with proper transfusion and treatment of his AVM (Figure 1B). His transthoracic echocardiography (TTE) revealed mild left ventricular hypertrophy and normal left ventricular ejection fraction (LVEF). The patient did well and was discharged home with a follow-up appointment for an outpatient visit for potential ischemic workup when GI condition permits.

Patient 2

A 57-year-old woman with a history of mild-intermittent asthma was presented to the ED due to a panic attack associated with substernal chest pain. In the midst of her panic attack, she complained of the development of substernal chest pressure. She had no other symptoms such as shortness of breath and syncope. She is a current tobacco smoker and utilizes amphetamine-based illicit drugs. She reported the use of amphetamine-based illicit drugs a few hours prior to presenting to the emergency room. Vitals showed a heart rate of 107 beats per minute and regular rhythm. The rest of her vitals were within normal limits and there were no significant findings on physical examination. 

**Figure 2:**
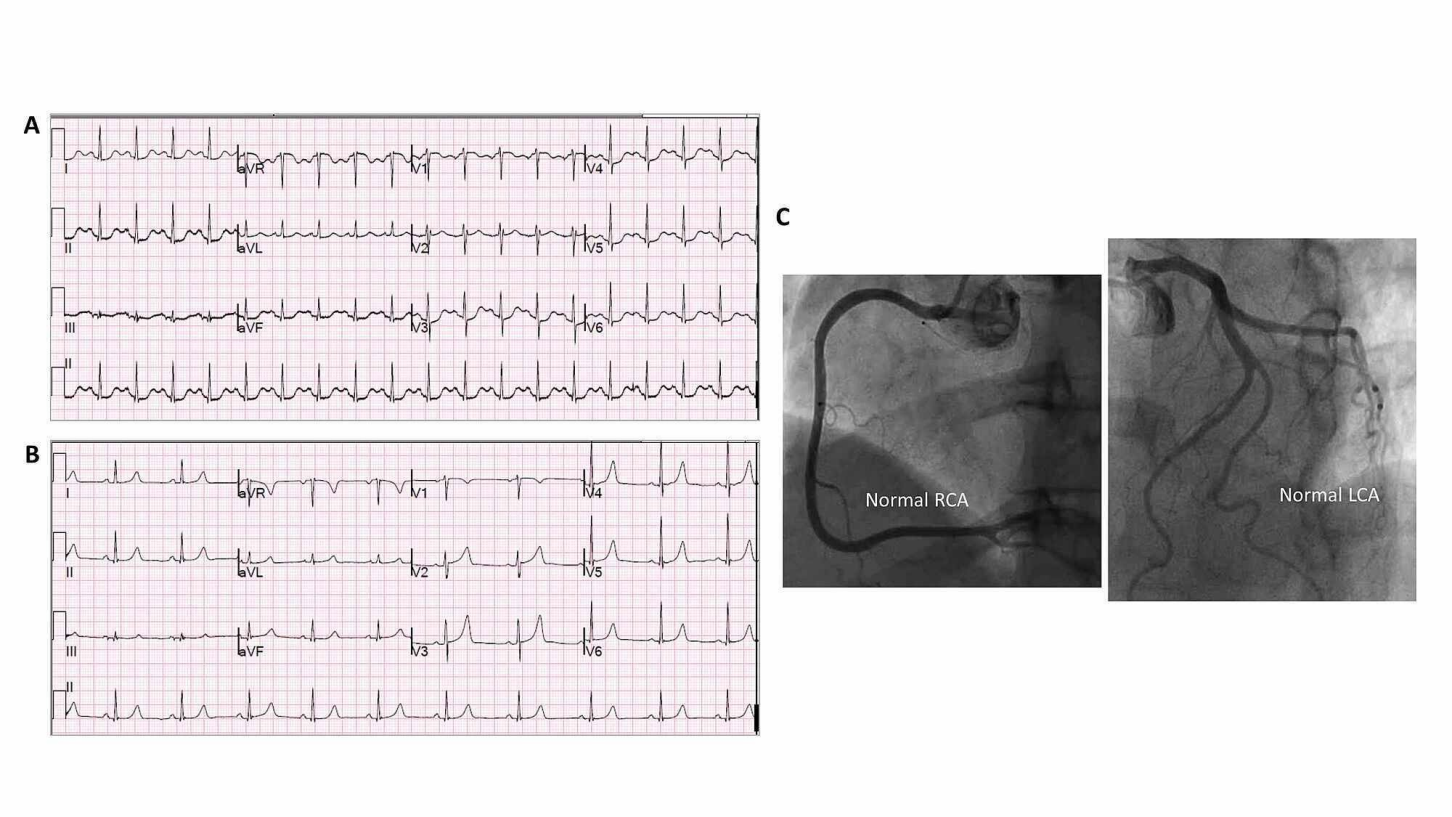
Twelve-lead ECG and coronary angiography of patient 2 (A) Presenting ECG showed sinus tachycardia with mild ST elevation in aVR and V1, and diffuse ST depressions in I, II, aVL, V3-V6. (B) Subsequent ECG after the resolution of symptoms showed normal sinus rhythm with no ST-T changes. (C) Coronary angiography shows normal coronary vasculature.

Her 12-lead ECG in the ED showed 1 mm STE in aVR and <1 mm STE in V1 with ST-segment depressions in seven different leads (Figure 2A). STEMI alert was activated in the ED. While the patient continued to be anxious, the status of her overall clinical condition was stable without any acute distress and her chest pressure slightly improved. Since her clinical presentation was atypical for myocardial infarction, the status of her overall clinical condition was stable, the ECG was non-diagnostic for STEMI, and the CCL activation was cancelled. Her urine drug screen was subsequently found to be positive for amphetamine and methamphetamine. Her troponin-T was initially elevated at 0.046 ng/ml (normal <0.03 ng/ml) and the subsequent two draws six hours apart were negative. Her D-dimer was negative and her basic metabolic panel was within the normal range. She was treated symptomatically overnight without further complaints. Her follow-up ECG showed resolution of the ischemic changes (Figure 2B). The coronary angiography showed normal coronaries (Figure 2C) and both her TTE and left ventriculogram revealed normal LVEF. The patient did not have any further episodes of chest pain and remained symptom-free upon discharge. 

Patient 3

**Figure 3:**
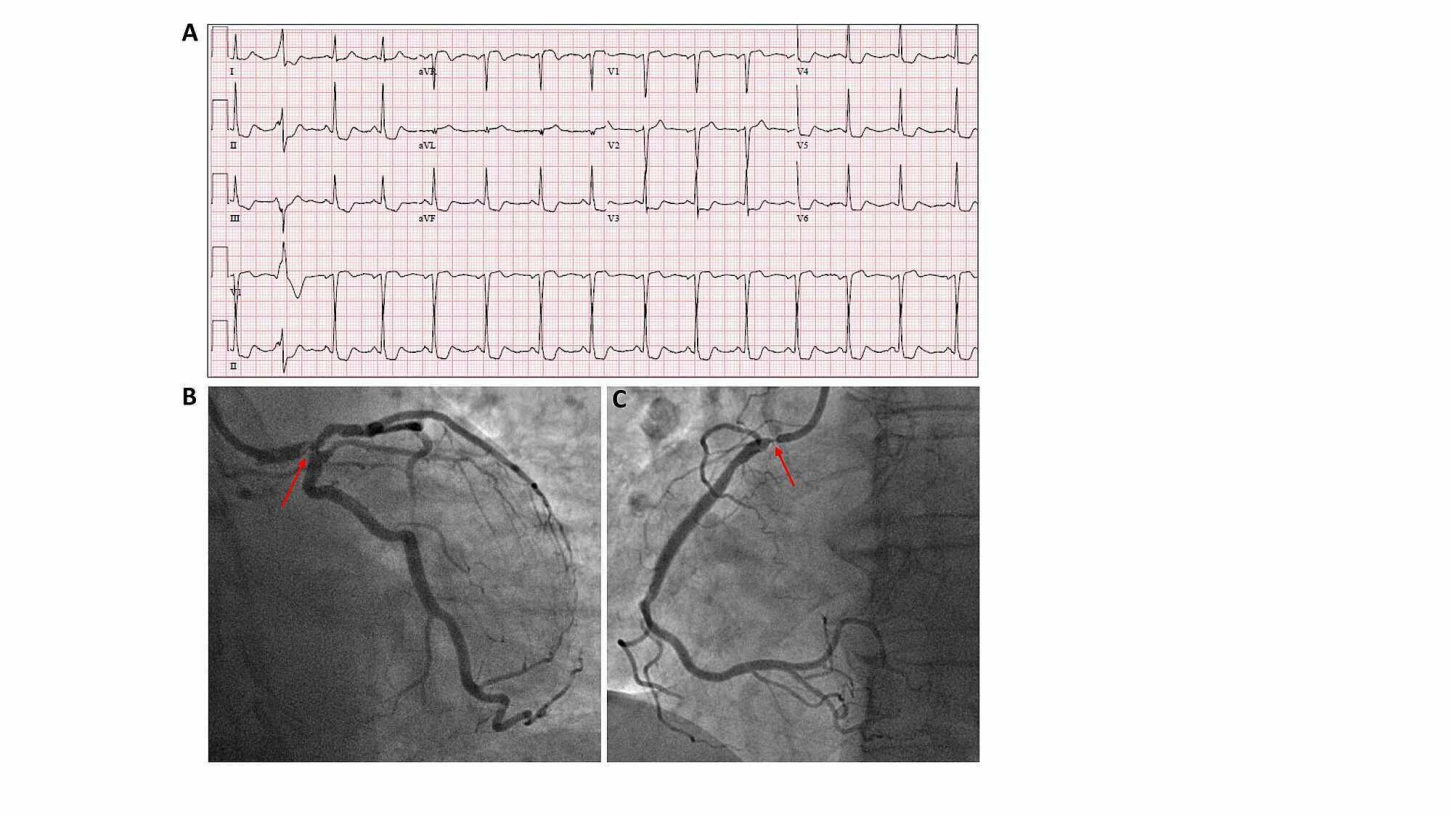
Twelve-ECG (A) and coronary angiography (B and C) of patient 3 (A) Presenting 12-ECG showed normal sinus rhythm with 2 mm STE elevation in aVR, 1 mm STE in V1, ST depressions in I, II, III, aVF, V3-V6. (B) LCA angiography shows 95% stenosis (red arrow) in the distal left-main. (C) 95% ostial RCA stenosis (red arrow). STE: ST-segment elevation, ECG: electrocardiogram, LCA: left coronary artery, RCA: right coronary artery.

A 68-year-old female with a history of heart murmur of unclear significance since childhood presented with a one-month history of burning epigastric pain. The symptoms usually occurred with meals; however, a trial of proton pump inhibitor, famotidine, and sucralfate failed to improve her symptoms. She lost about 15-20 pounds over the previous month due to poor oral intake. Physical examination showed a pulse rate of 87 beats per minute, a blood pressure of 110/72 mmHg, a respiration rate of 18 respirations per minute, and oxygen saturation of 95% breathing ambient air. A cardiac examination revealed an I/VI systolic murmur at the right upper sternal border and left lower sternal border. The rest of her physical examination was unremarkable. Initial laboratory testing in the ED revealed elevated cardiac troponin-I at 1.4 ng/ml (normal <0.3 ng/ml). The hemoglobin was 13.8 g/dL, hematocrit 41.8%, BUN 11 mg/dL, and creatinine 0.73 mg/dL. Her 12-lead ECG in the ED showed 2 mm STE in aVR, 1 mm STE in V1, and horizontal ST depressions in I, II, III, aVF, and V3-V6 (Figure 3A). She was subsequently admitted for a diagnosis of non-ST-elevation myocardial infarction (NSTEMI). A cardiologist was consulted and an urgent coronary angiography was performed revealing a 95% distal left main stenosis (Figure 3B; Videos 1 and 2) and a 95% ostial right coronary artery (RCA) stenosis (Figure 3C; Video 3). She underwent emergent coronary artery bypass grafting with the left internal mammary artery (LIMA) to left anterior descending (LAD), reverse saphenous vein graft (SVG) to RCA, and SVG to left circumflex artery (LCx). The patient had an uneventful post-op recovery and her post-op TTE revealed an LVEF of 35-40%. She is doing well and continues to follow-up in our cardiology outpatient clinic.

**Video 1:** LCA angiography (RAO/CAU projection) Severe eccentric stenosis visualized in the distal left-main. The left circumflex and left anterior descending arteries have diffuse luminal irregularities without significant stenosis. LCA: left coronary artery, RAO/CAU: right anterior oblique caudal view.

**Video 2:** LCA angiography (LAO/CRA projection) Severe eccentric stenosis in the distal left-main is consistent with Video 1. LCA: left coronary artery, LAO/CRA: left anterior oblique cranial view.

**Video 3:** Right coronary artery angiography (LAO to RAO rolling projection) Severe ostial RCA stenosis is visualized. RCA: right coronary artery, LAO: left anterior oblique, RAO: right anterior oblique.

## Discussion

The common clinical feature of these cases is the presence of significant STEs in lead aVR with associated horizontal ST-segment depressions in multiple infero-lateral leads (I, II, III, aVL, aVF, and V3-V6). This pattern of ECG is widely recognized by clinicians as an indicator for global myocardial ischemia. The subsequent workup from this case series revealed vastly different etiologies that would explain these particular ECG findings, such as severe anemia (patient 1), tachyarrhythmia, and possible coronary vasospasms due to toxic drug overdose (methamphetamine and amphetamine; patient 2), and most importantly, severe left main and multi-vessel coronary artery disease (CAD) requiring emergent bypass surgery (patient 3). While STE in lead aVR is non-specific for any one condition [4], it is a reliable indicator for global myocardial ischemia and should be considered a high-risk feature prompting immediate attention.

Since lead aVR reflects the myocardial perfusion of the basal septum, which is often affected by the LMCA or ostial/proximal LAD, it has gained significance in detecting acute myocardial ischemia. Due to the high mortality rates of these lesions, there has been an extensive examination of applying STE in aVR as a diagnostic and prognostic tool for these high-risk lesions [5-7]. The addition of diffuse ST-segment depressions in eight or more leads to the STE in aVR was found to have a predictive accuracy of 75% for LMCA or triple vessel disease [5,8]. The degree of STE (>0.5 mm vs <0.5 mm) in lead aVR was associated with significantly different predictive values for left main or multi-vessel CAD in patients with the acute coronary syndrome (ACS), specifically STE > 0.5 mm in lead aVR significantly predicted high risk of having LMCA or multi-vessel CAD in a recent meta-analysis [7]. Knotts et al. reported a 43% rate of coronary angiography (57 patients) as part of the evaluation for patients who presented to the ED with ECG findings of STE in lead aVR and ST-segment depressions in over seven leads [9]. Among the 57 patients who underwent coronary angiography, only 40% (17% of the total patients with aVR STE and >7 leads with ST-segment depressions) were found to have LMCA, left main equivalent, or multi-vessel CAD.

On the other hand, Knotts et al. reported only approximately one third (28%) of patients who presented with STE in aVR were due to ACS; the other third of patients were related to tachyarrhythmia and the last third of patients were due to non-cardiac shocks such as septic shock, hemorrhagic shock, or aortic dissection [9]. Our clinical experience and review of the literature revealed a list of potential clinical etiologies for an ECG presentation with STE in aVR and diffuse ST depressions (>6 leads; Table 1). Anticoagulation and antithrombotic therapy are contraindicated in some of these etiologies; however, they are essential for some of the other etiologies. Therefore, in clinical practice, when evaluating high-risk patients such as our patients with STE in aVR and diffuse ST-segment depressions, the immediate decision regarding the workup approach and treatment strategy is critical especially in regards to anticoagulation and antiplatelet therapy. Therefore, we categorized the potential clinical etiologies in Table 1 in a manner that is helpful in guiding the decision to initiate anticoagulation and antithrombotic (A.C.) therapy.

**Table 1:** Role of A.C. therapy in all the potential clinical etiologies for aVR STE and diffuse ST depression on ECG *Hemodynamic unstable or potentially unstable; A.C.: anticoagulation or antithrombotic therapy, SVT: supraventricular tachycardia, STE: ST-segment elevation, ACS: acute coronary syndrome, ECG: electrocardiogram, CAD: coronary artery disease.

A.C. Indicated or Likely Indicated	A.C. Contraindicated	A.C. Not Immediately Required
ACS with potential left main or multi-vessel CAD decompensated HF (Killip Class III-IV) with or without CAD or ACS cardiogenic shock* massive pulmonary embolism*	Severe anemia due to bleed shock/hypotension due to sepsis or hemorrhage* aortic dissection* acute pericarditis	Significant tachyarrhythmia (i.e., Brugada syndrome, re-entry tachycardia, SVT) tachycardia in the setting of severe left ventricular hypertrophy vasospastic agent overdose severe aortic stenosis* electrolytes imbalance (i.e., hypokalemia)

Maintaining a broad differential for STE in aVR with or without diffuse ST-segment depressions is critical. It could have a significant clinical impact on the selection of time-sensitive workup and treatment strategies, which often involve activating the CCL and subjecting patients to invasive procedures, highly potent antithrombotic and anticoagulant therapy, and advance therapies for hemodynamic support. While there is an abundance of literature highlighting the importance of STE in lead aVR with ST-segment depressions in predicting high-risk ischemic conditions, our case series highlights the importance for clinicians to have a high level of awareness of the following facts: (1) less than a quarter of all patients who presented with STE in lead aVR were found to have severe ischemic heart condition [9]; (2) a significant portion of these patients may have contraindications to or not require potent anticoagulation or antithrombotic therapy (Table 1); and (3) the combined presence of conditions that require anticoagulation and conditions where anticoagulation are contraindicated in a single patient. The importance of understanding these concepts could help decrease false CCL activations, decrease the risk of subjecting patients to high-risk medications, and decrease the number of unnecessary invasive procedures and their associated risks. For instance, in patient 1, an emergent left heart catheterization and the associated use of periprocedural anticoagulation would have exacerbated his gastrointestinal bleeding leading to worse outcomes. 

**Figure 4:**
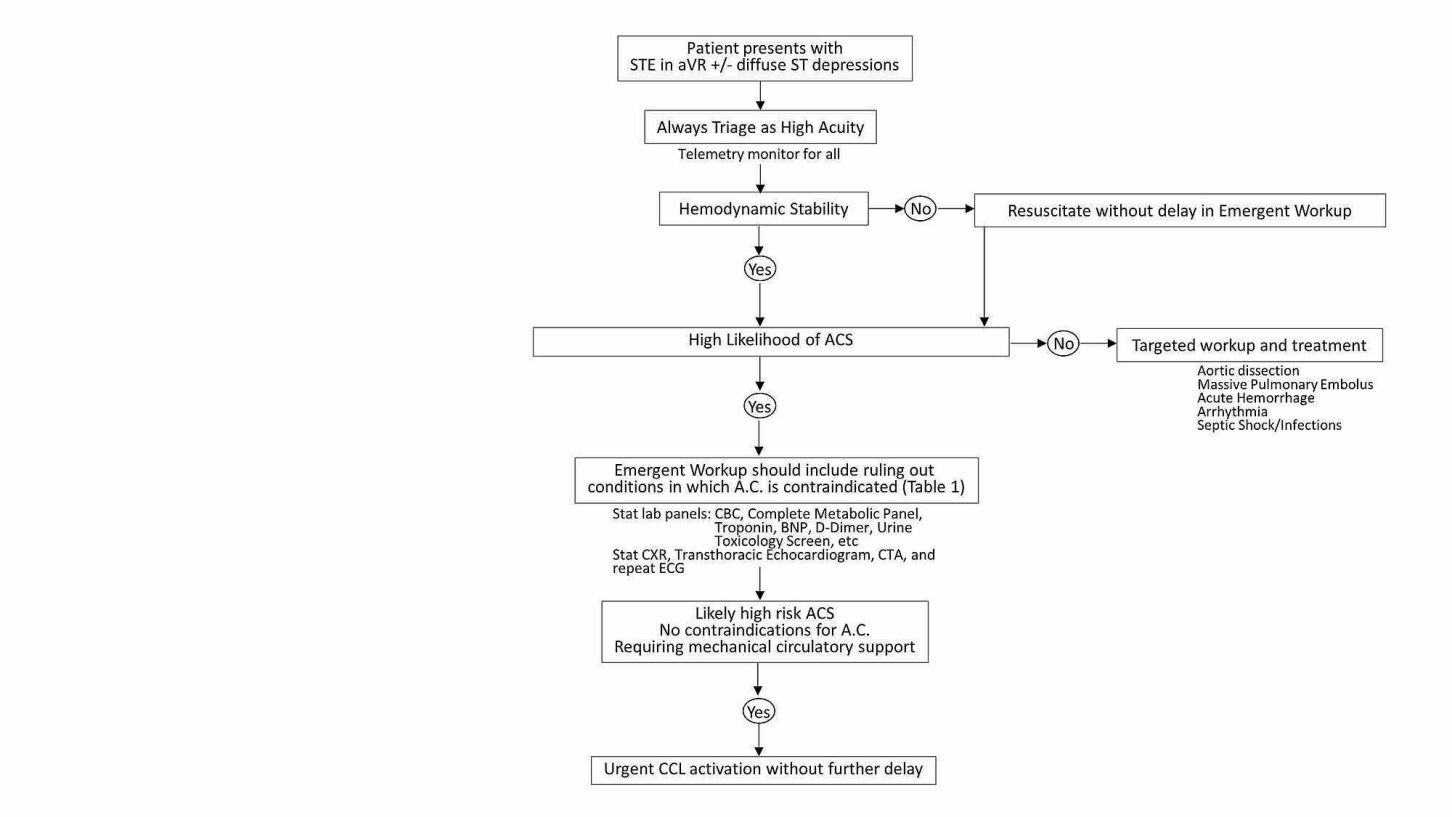
Proposed systemic approach to evaluate patients presenting with STE in lead aVR with or without associated diffuse ST depressions on ECG STE: ST-segment elevation, ECG: electrocardiogram.

Based on the currently available evidence and the concepts presented in this case series, we propose a clinical decision making flow chart in patients who present with STE in lead aVR with or without associated diffuse ST-segment depressions as depicted in Figure 4. All patients with these ECG findings should be triaged as high risk and should prompt continual telemetry monitor and immediate workup. Hemodynamically unstable patients should be aggressively resuscitated and at the same time, emergent workup should be continued without delay. Hemodynamically stable patients should have an emergent workup to guide the clinical decision on a therapeutic therapy within a timely manner. Emergent workup should include a broad array of screening to rule out (a) conditions such as severe anemia, active bleeding, aortic dissection (if clinically indicated by subjective history and physical examination), etc., in which anticoagulation and antithrombotic therapy are detrimental and (b) non-ACS conditions such as severe electrolyte disturbances due to renal failure, critical aortic stenosis, decompensated heart failure, etc. Resonating with the Hippocratic Oath in which, physicians should “first do no harm,” such an approach to patients with STE in aVR with or without diffuse ST-segment depressions would provide the necessary information to safely treat patients in a timely manner for possible high-risk ACS with global ischemia likely due to LMCA, left-main equivalent, or multivessel CAD. 

## Conclusions

STE in lead aVR with diffuse ST depressions (in ³6 leads) reflects global myocardial ischemia. While in the clinical setting of an ACS, this pattern was found to be associated with a high probability of significant left-main or multi-vessel CAD, a broad panel of other etiologies could also lead to such an ECG pattern. In many of these potential etiologies, anticoagulation or antithrombotic therapy which are commonly applied in ACS management and invasive CCL procedure, are detrimental or contraindicated. Our case series highlighted the need for a systematic approach in evaluating and managing patients with STE in aVR with emphasis on the safety of our patients.
